# Late Cretaceous coprolite from the Opole area (southern Poland) as evidence for a variable diet in shell-crushing shark *Ptychodus* (Elasmobranchii: Ptychodontidae)

**DOI:** 10.7717/peerj.16598

**Published:** 2023-12-15

**Authors:** Dawid Mazurek, Mateusz Antczak

**Affiliations:** 1 Institute of Biology, University of Opole, Opole, Polska; 2 European Centre of Palaeontology, University of Opole, Opole, Poland

**Keywords:** Fossil, Coprolite, Chondrichthyes, Shark, Palaeoecology

## Abstract

**Background:**

Coprolites, *i.e*., fossilized faeces, are an important source of knowledge on the diet and food processing mechanisms in the fossil record. Direct and indirect evidences for the dietary preferences of extinct sharks are rare in the fossil record. The first coprolite attributable to *Ptychodus* containing prey remains from the European Cretaceous is documented here.

**Methods:**

A coprolite from the Late Cretaceous of Opole (southern Poland) was scanned using micro-computed tomography to show the arrangement of the inclusions. In addition, the cross-section was examined under the SEM/EDS to analyse the microstructure and chemical composition of the inclusions.

**Results:**

Brachiopod shell fragments and foraminiferan shells are recognized and identified among the variously shaped inclusions detected through the performed analysis.

**Conclusions:**

The extinct shell-crushing shark *Ptychodus* has been identified as the likely producer of the examined coprolite. The presence of brachiopod shell fragments indicates that at least some species of this durophagous predatory shark may have preyed on small benthic elements on the sea bottom.

## Introduction

Coprolites, *i.e*., fossilized faeces, together with consumulites (intestine contents), gastroliths (stomach, or gizzard, stones), and regurgitalites (orally expelled masses) make up the group of ichnofossils known as bromalites (Hunt & Lucas, 2021). These are informative for establishing the diet and food processing style. The major caveat is the uncertainty concerning the specific producer of this kind of fossils. Sometimes, the co-occurrence in the same strata of fossils and faeces, and specific features of the animal linking the coprolite and skeletal material (*e.g*., size, purported diet), can be used as means to pinpoint, with a certain level of certainty, the most likely producer. This was done for the Late Triassic site of Krasiejów in the Opole area, where small coprolites, containing insect remains, were identified as a product of a co-occurring dinosauromorph *Silesaurus opolensis*, with the main reasoning based on body sizes and possible diets of the skeletally identified fauna at this locality (Qvarnström et al., 2017, 2019, 2021). The discussion there, however, did not take into account a range of taxa from the site identified thus far only on the basis of dental remains. Shark teeth and coprolites are a common find in Late Cretaceous deposits, including the Turonian-Coniacian of Opole area (Mazurek, 2008). Skeletal fossils consist mainly of isolated teeth, with few finds of an associated dentition or even a single vertebra (pers. obs.). Niedźwiedzki (2005) and Niedźwiedzki & Kalina (2003) are the only authors that have studied the shark fauna of the Opole area in recent years. Niedźwiedzki & Kalina (2003) described from Opole the following taxa: *Ptychodus latissimus*, *P. mammillaris*, *P. polygyrus*, *Squalicorax* sp., *Scapanorhynchus raphiodon*, and *Paranomotodon angustidens*. Niedźwiedzki (2005) listed jointly taxa from localities at Opole and Sudetes area. Apart from those mentioned above, other taxa said to be common were *Cretoxyrhina mantelli*, *Cretolamna appendiculata*, *Squalicorax falcatus*, and *Odontaspis subulate*, while rare finds included *Hexanchus microdon*, *Synechodus major* and *Hybodus dentalus*. In a popular book (Yazykova, 2022), Niedźwiedzki confirms the presence specifically in the Opole area of *Squalicorax falcatus*, *Cretolamna appendiculata*, *Cretoxyrhina mantelli*, and *Odontaspis subulata*. These works are supplemented by the collecting efforts of the current authors, whose rich collection preserves *Squalicorax falcatus* and other lamniforms, *Ptychodus* spp., as well as a single find of a hexanchiform.

As for coprolites, spiral shark faeces are especially common in clayey marls. Their general presence was already noted by Mazurek (2008) and Hunt et al. (2015). Here, we present and document in detail for the first time one of the coprolites from the Upper Cretaceous of Opole (southern Poland). The specimen was analysed by SEM-EDS and microCT to investigate structure and chemical composition of the inclusions. Based on the shape and prey content of the coprolite and the dietary preferences of the co-occurring ichthyofauna, the coprolite producer was identified and its behaviour was discussed in a palaeoecological context.

## Geological setting

Odra II quarry is a working quarry within the city of Opole (southern Poland). The exposed rock sequence starts with clayey marls (Middle Turonian *Inoceramus apicalis* Zone) and proceeds with limy marlstones (Middle Turonian *I. lamarcki* Zone to the lowermost part of Upper Turonian *I. perplexus* Zone), and ends with marly limestones (*I. perplexus* Zone). This sequence of strata forms part of a single transgression-regression megacycle (Cenomanian-Coniacian) that represents the Cretaceous strata of the so-called Opole Trough (Jagt-Yazykova et al., 2022). The biota preserved is numerous and consists of ichnofossils, sponges, inoceramids and other bivalves, brachiopods, fish remains, cephalopods, echinoderms, crustaceans, cnidarians, shark coprolites, land flora, and rare marine reptiles. The coprolites are quite common and of uniform size and shape, with spiral structure pointing to sharks (Dentzien-Dias et al., 2012). The specimen studied comes from the clayey marls (Middle Turonian: *I. apicalis* Zone).

## Materials and Methods

A coprolite was collected from the Odra II quarry (Oleska street, Opole) during the summer digging camp in 2020. It is housed at University of Opole (col. no. IBUO-DM-KOPRO1). Fieldwork was possible due to the legal agreement between the quarry owner (Cement Factory “Odra”) and European Centre of Palaeontology, University of Opole dated 24.05.2017.

**Figure 1:**
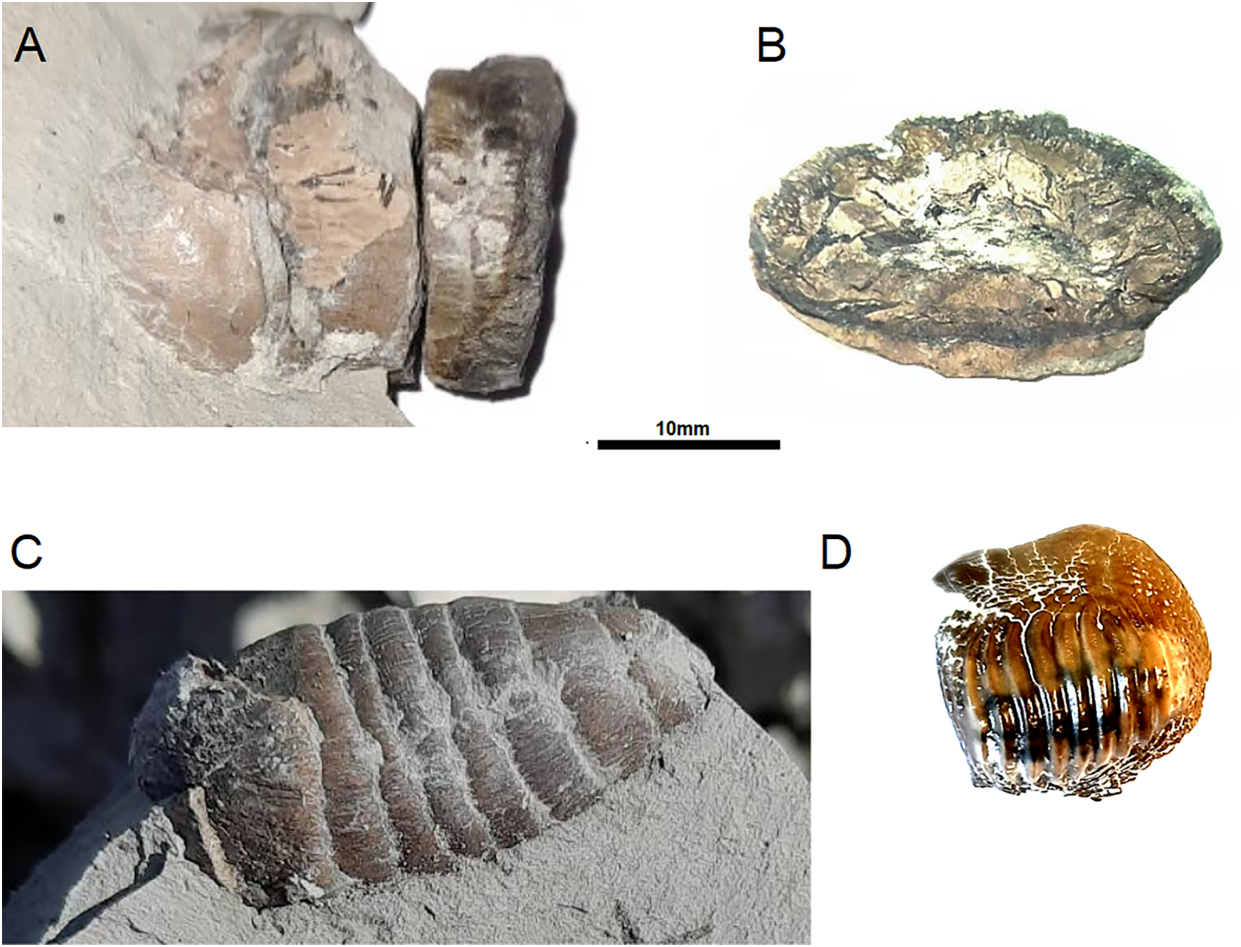
*Ptychodus* remains from opole cretaceous. Analysed coprolite IBUO-DM-KOPRO1 in lateral view (A) and cross-section (B). Coprolite IBUO-DM-KOPRO2 in lateral view (C). Teeth IBUO-DM-ZAB1 (D).

The coprolite is incomplete and the preserved portion is 22 mm in length. The estimated size of the coprolite could be at least two times larger compared to other specimens in the collection ranging between ca. 20–55 mm in total length. As the specimen is broken, some dark infillings are visible within the grey phosphatic mass on the cross-section (Fig. 1). To determine the composition of the infilling, the specimen was analysed with micro CT scanner SkyScan 1273 in Bruker Laboratory in Kontich, Belgium. Obtained data were presented using DataViewer (for multiple cross sections in three directions) and CTVox (for the presentation of the 3D orientation of infillings) software. A 8.5 µm resolution scan is available in the Morphosource database (https://doi.org/10.17602/M2/M514300) in the form of 2,882 TIFF image series.

For chemical identification of the infilling, the surface of the broken part (cross-section) was polished with grinding powder. The obtained polished surface was examined under Scanning Electron Microscope TM 3000 with secondary electrons as well as with the use of Energy-Dispersive X-ray Spectroscopy. In addition, the coprolite IBUODM-KOPRO2 (Fig. 1C) was selected as comparison material.

## Results

**Figure 2:**
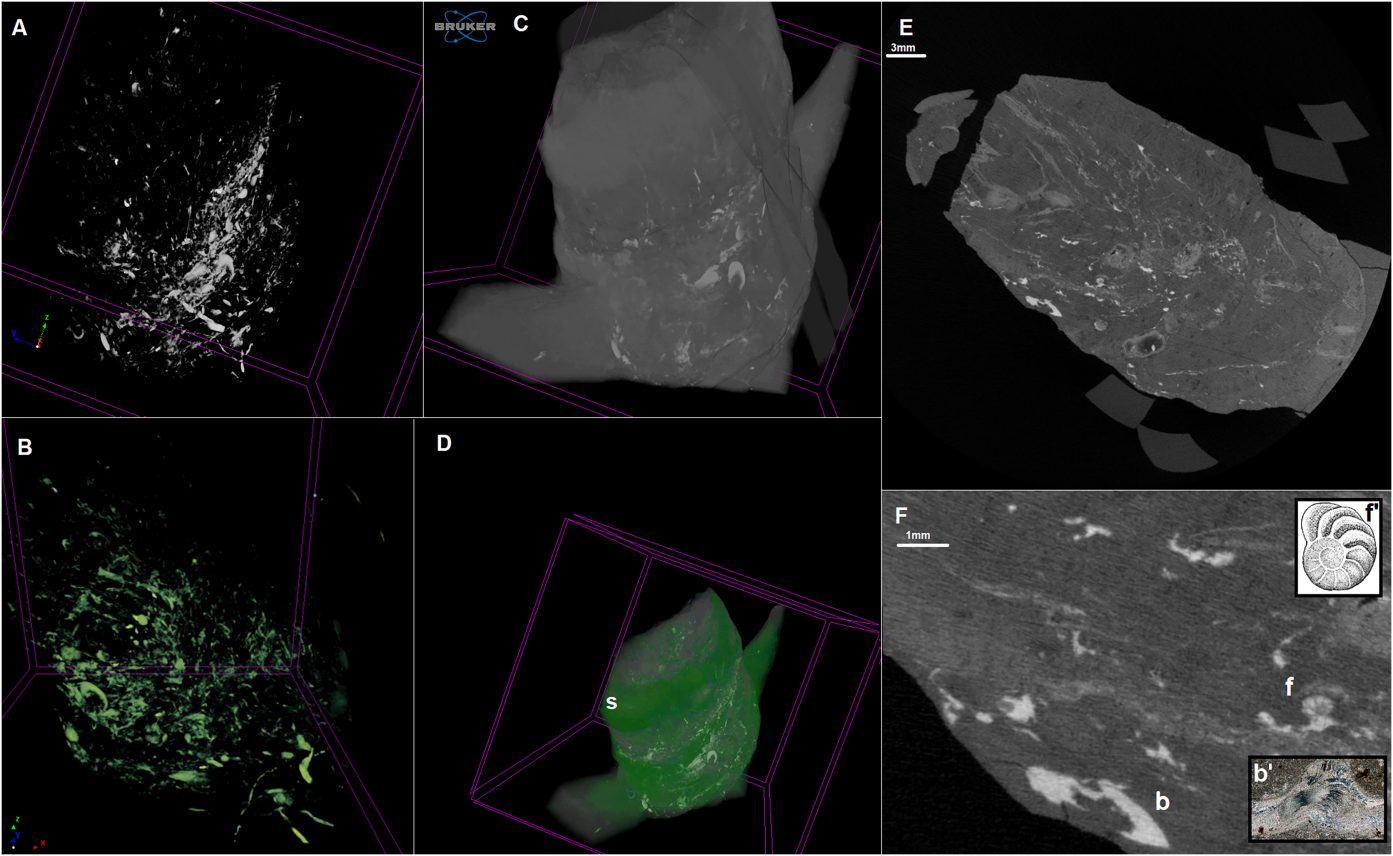
MicroCT scan of the coprolite. Infillings–3D model (A and B). Coprolite mass with infillings–3D model (C and D). Longitudinal cross-section (E and F). b, brachiopod shell; f, foram shell. S, spiral structure. *Gavelienella* illustration from Hornibrook, Brazier & Strong (1989), Fig. 18.17. Brachiopod shell photograph from alexstrekeisen.it. 3D model made in CTVox. Scan resolution: 8.5 µm. Image credit: https://pal.gns.cri.nz/foraminifera/www/HBS362.htm, © Copyright in 2018 by GNS Science and is licenced for re-use under a Creative Commons Attribution 4.0 International License.

**Figure 3:**
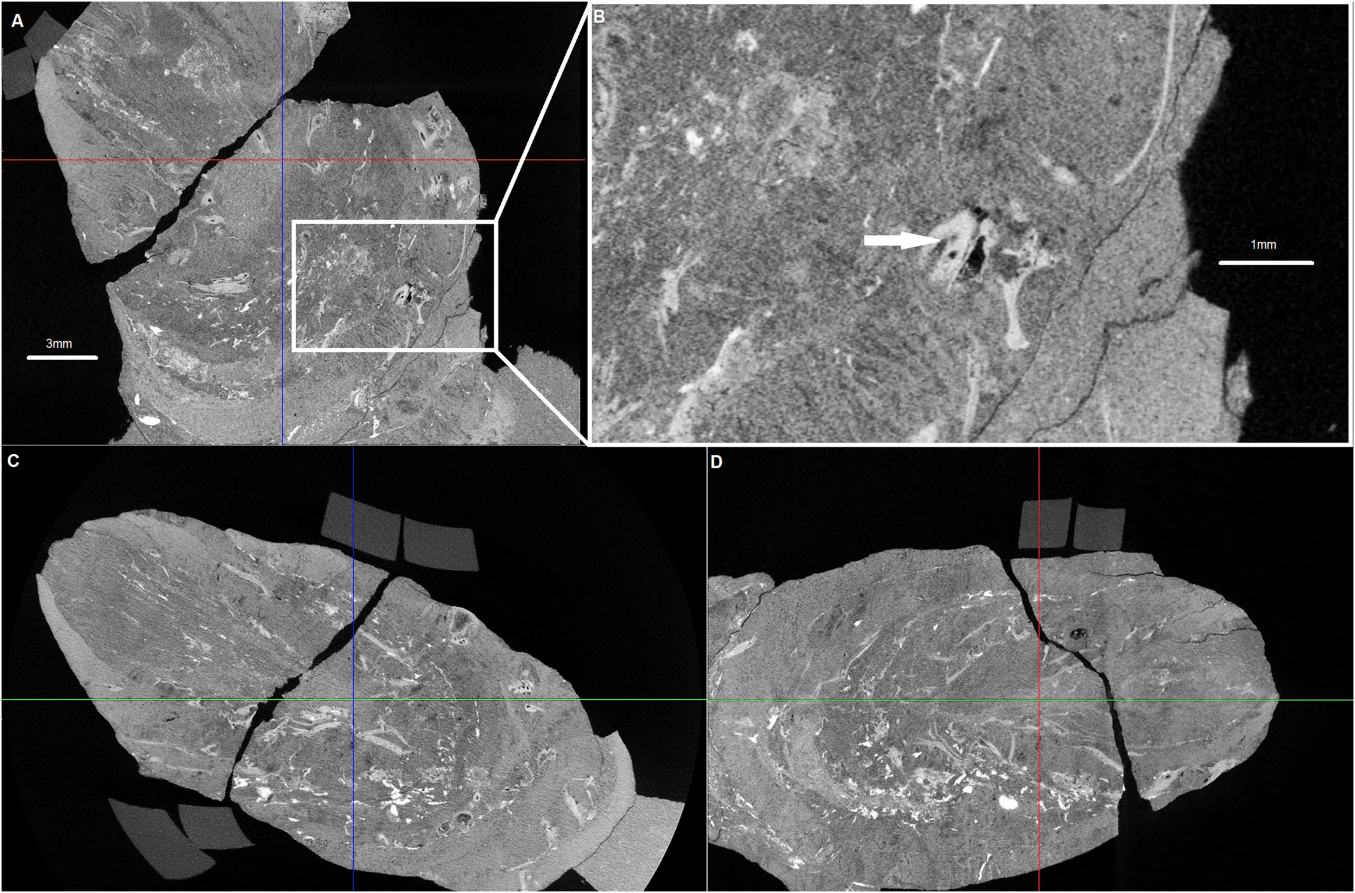
Cross sections of the analysed coprolite. Cross-sections of the analysed coprolite in three directions (A, C and D). Magnification of the example of indet. shell fragment (B). Image obtained in DataViewer.

**Figure 4:**
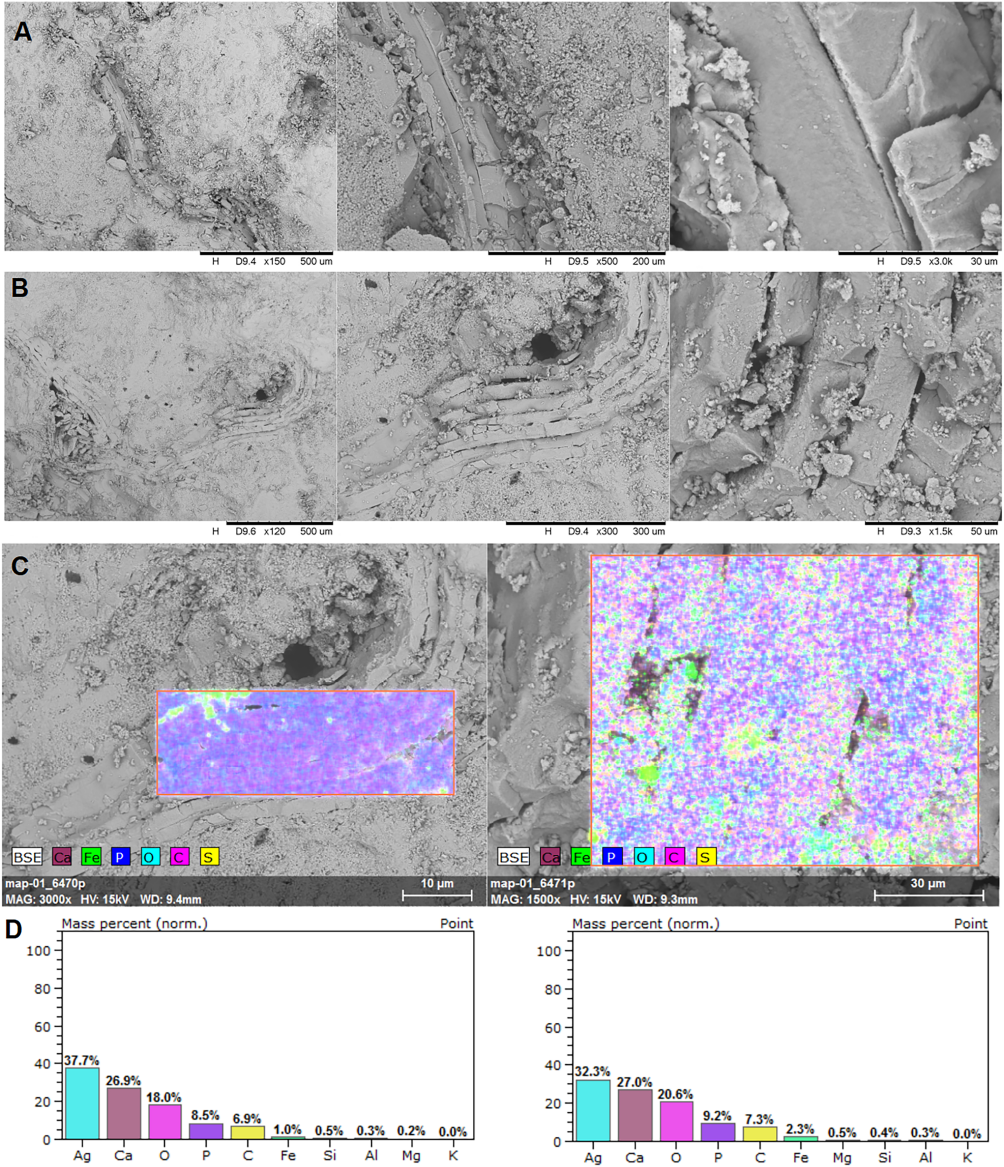
EDS analysis. Brachiopod shell fragments (A and B), the surface of the EDS analysis (C), and mass percentage result (D). SEM photographs: the authors. They were made at Faculty of Chemistry, University of Opole, Opole, Poland.

Examined specimen and additional IBUO-DM-KOPRO2 possess a heteropolar spiral shape (see also Fig. 2D), which is typical of chondrichthyan coprolites (see Eriksson et al., 2011; Dentzien-Dias et al., 2012). MicroCT scans reveals numerous infillings with densities differing from the phosphatic matrix of the coprolite (Figs. 2 and 3). Most of the shapes are irregular, many being boat-shaped. Some of them can be recognized and assigned to certain groups of animals, specifically micromorphic brachiopods (Fig. 4) and foraminifera (Fig. 2F), based on, SEM observations of microstructure and cross-section visible in micro CT scan. Two unidentified shells/tests have been observed under higher magnification under SEM. Both inclusions (Fig. 4) show the walls consisting of horizontal lamellae. No vertical elements are present, which would be expected in the case of an inoceramid prismatic layer (*e.g*., Jiménez-Berrocoso, Olivero & Elorza, 2006), one of the possible prey. No macroscopic chunks of large bivalves are present either. The microstructure is more reminiscent of an inpuncate brachiopod shells (Griesshaber et al., 2007). Regardless, some inclusions are firmly identified as brachiopods and forams (Figs. 2 and 3), while no traces of other possible shelled (*e.g*., inoceramids, see Hattin, 1975) or soft-bodied prey were detected.

In the EDS analysis, the main elements are Ca, O, C, and P (Fig. 4).

## Discussion

Irregular and boat-shaped infilling creates a specific pattern. Similar infillings can be observed in coprolites of durophagous fishes from the Middle Triassic (Antczak et al., 2020). EDS signature suggests that these are elements made of calcium carbonate. The matrix of the coprolite possesses a phosphatic character. The spiral heteropolar nature of the Opole Cretaceous coprolites points to sharks as their producers. Taking into account the above, it strongly suggests that the analysed coprolite was produced not by a piscivorous shark but rather by species feeding on invertebrates with calcareous shells. The only known candidate is *Ptychodus*. Currently, this genus is thought to be a facultative durophage, with diet composed of inoceramids and other shelly fauna, but also fishes (Shimada, Rigsby & Kim, 2009; Amadori et al., 2019, 2020, 2023; Hamm, 2020). The assignment of some of the infillings to brachiopods suggests that the producer was feeding at the bottom of the sea (nektobenthonic) instead of in open water (nektonic). In addition, tests of calcareous foraminifera can be recognized, similar to genera *Lenticulina* or *Gavelinella* (Kłapciński & Teisseyre, 1981; Strong, Raine & Terezow, 2018) which are bottom-dwelling taxa, probably swallowed accidentally together with the sediment and a brachiopod laying on the bottom of the sea.

**Figure 5:**
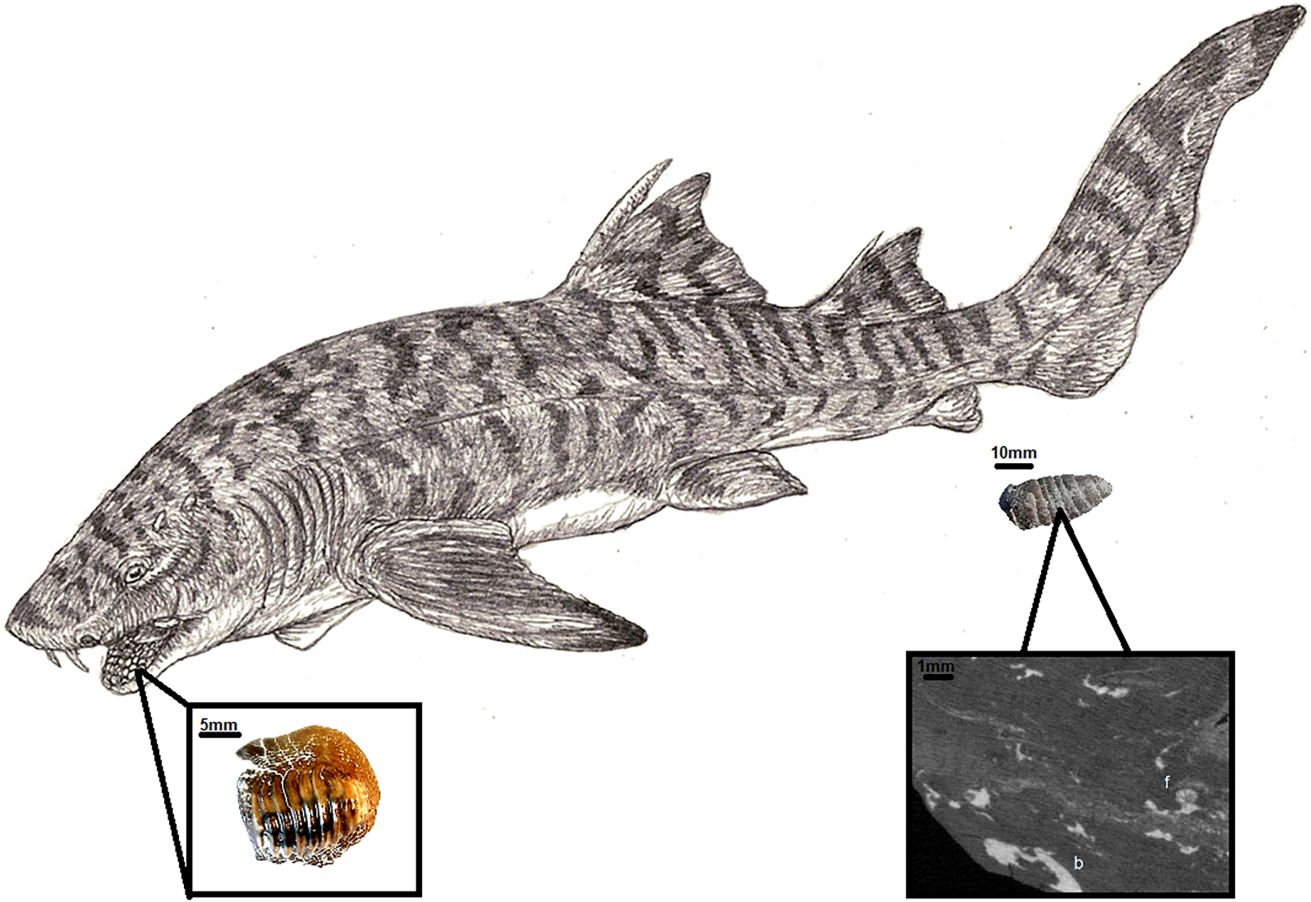
*Ptychodus* reconstruction (Author: Jakub Kowalski) with an example of tooth IBUO-DM-ZAB1 and coprolite (IBUO-DM-KOPRO2). Magnification of the internal structure of the coprolite comes from IBUO-DM-KOPRO1.

In the Turonian of Opole, several shark species could produce coprolites of this size. These include: *Cretoxyrinha, Scapanorhynchus, Hexanchus, Squalicorax*, and *Ptychodus*. Among them, only the last is commonly described as durophagous based on tooth morphology (Shimada, Rigsby & Kim, 2009; Shimada et al., 2010; Amadori et al., 2022) (Fig. 5). Niedźwiedzki & Kalina (2003) identified at the Opole Cretaceous three taxa of *Ptychodus*. Apart from isolated teeth, the Opole Cretaceous also yielded two sets of teeth: one is deposited at the University of Wrocław (MGUWr, unnumbered), while the other is in collection of the University of Opole (IBUO-DM, unnumbered). Similar finds are known for several taxa worldwide (Amadori et al., 2019; Hamm, 2017), with partial skeletons or skulls much rarer (Shimada, Rigsby & Kim, 2009; Shimada et al., 2010).

The occurrence of *Ptychodus* as the only durophagous shark suggests that the producer of the coprolite might be specifically identified to the mentioned genus. However, the lack of bivalve shell fragments within the coprolite is notable. There are several possible explanations.

The first possibility is that the producer of the coprolite fed also on the common inoceramids, but was able to feed only on the soft tissue and for example orally reject the hard shells. The modern mammal *Odobenus rosmaris* feeds on benthic mollusks by sucking the soft tissue and ejecting the hard parts (Scheyer et al., 2011). However, currently, no dentalites were recognized from Opole Cretaceous inoceramid shells (even though many microscopical epifauna remnants can be observed—*e.g*., Bryozoa, Serpulidae, Ostreoida). From numerous specimens described by Walaszczyk (1992) a single sublethal injury was mentioned. If sharks were efficient predators we would predict evidence of failed prey subjugation. However deformations and growth iterations in inoceramid shells are known, they are, rather, effects of decapod predation (Harries & Ozanne, 1998). Of note, none of the coprolites we studied externally seem to contain any large shelly material. To the best of our knowledge, none are known elsewhere.

The second possibility is that the fossils of a coprolite producer are not present (or not recognized yet) in the Quarry due to the sedimentation bias or being less common representative of the Cretaceous fauna of this area. Hunt et al. (2015) show that producers of coprolites are often not represented by body fossils. Chondrichthyan fossilized faeces are the most common, while in terms of body fossils palaeoichthyofaunas are usually much more diversified, which Hunt et al. (2015) termed the ‘shark surplus paradox’.

The third option, explaining this the lack of dentalites and brachiopod infillings in the described coprolite, is to consider *Ptychodus* (the form from Opole, and by extensions possibly also other members of the genus) as the producer which, contrary to some current opinions, was not a strictly durophagous taxon, but rather a durophagous-filter feeder specialized in small prey, with bulbous teeth for crushing shells, but also with water moving between the ridges of the teeth (Fig. 1) or more likely rejecting water and sediment through gills like modern myliobatiform rays that fluidize the sediment by means of jaws’ movement (Sasko et al., 2006). The sediment of the Cretaceous chalk seas might already be soupy in consistence and *Ptychodus* might sift it in search for small shelly fauna. Such elaborated ornamentation as present on the teeth of *Ptychodus* is lacking in many other durophagous taxa except skates, including among others: various fishes (*e.g*., Purnell & Darras, 2015; Raguin et al., 2020), placodonts (Pommery et al., 2021) and many mosasaurs (Leblanc, Mohr & Caldwell, 2019); the teeth are often restricted to the outer edge of the jaws, and supposed shark dentalites on inoceramids and other hard elements are rare in the literature known to us (*e.g*., Kauffman, 1972; Hunt & Lucas, 2021, Table A.5), which, however, can be ascribed to poor taphonomic potential of such finds, and the lack of both recognition and studies devoted to them. Also, not all filter-feeders possess small, gracile, sieve-like teeth. Several species of pinnipeds have teeth modified into filter-feeding, specifically with elaborate cusps of postcanines on both the upper and lower jaw. This modification is well-seen, especially in the crabeater seal *Carinophaga lobodon* (Chatterjee & Small, 1989; Bengtson, 2002; Adam, 2005).

## Conclusions

MicroCT scan and EDS analysis show that coprolite collected in the Turonian deposits of Odra II quarry in Opole, southern Poland is filled with shell fragments. Inclusions can be identified as remains of small brachiopods (and occasionally Foraminifera). Such content suggest that the producer’s diet was based on the small shell-covered organisms encased within the sediment, possibly revealing mix of durophagy and filter-feeding strategy, *i.e*., a process of sifting the sediment first and then crushing the remaining fraction. According to the shape of the coprolite, it can be described as belonging to shark. Within chondrichthyan fauna of the locality, there is only one species of durophagous shark, *Ptychodus*; thus, it can be proposed as the likely producer of the analysed coprolite, although the impact of the ‘shark surplus paradox’ (the high diversity of ichthyofaunas contrasting with a low diversity of coprolite ichnofaunas in Cretaceous chalk facies) cannot be entirely ruled out (Hunt et al., 2015).

*Ptychodus* (if considered a producer) might have been a durophagous-filter feeder (partially analogous to modern myliobatiforms feeding habit) and not a strictly durophagous fish as there is no evidence of preying on abundant large inoceramids and other common shelly organisms (in the forms of coprolites or regurgitalites). While we acknowledge this hypothesis cannot necessarily be universally applied to other species of the genus, or different growth stages—in the context of scarcity of direct evidence worldwide for preying on large shelly organisms, we tentatively suggest that some form of both durophagy and filter feeding ecology might need to be considered for *Ptychodus* spp. individuals. Further investigation of coprolites and, when available, gut contents will be necessary to confirm or reject the hypotheses proposed in this study.

## Additional Information and Declarations

### Field Study Permissions

The following information was supplied relating to field study approvals (*i.e*., approving body and any reference numbers): Field works were approved by the quarry owner on the rights of agreement between Odra S. A, and European Centre of Palaeontology (24.05.2017).

### Data Availability

The following information was supplied regarding data availability: The CT scan is available at Morphosource: https://doi.org/10.17602/M2/M514300. The physical specimen is housed at University of Opole palaeontological collection (IBUO-DMKOPRO1).

## References

[ref-1] Adam PJ (2005). Lobodon carcinophaga. Mammalian Species.

[ref-2] Amadori M, Amalfitano J, Giusberti L, Fornaciari E, Carnevale G, Kriwet J (2020). The Italian record of the cretaceous shark, *Ptychodus latissimus* Agassiz, 1835 (Chondrichthyes; Elasmobranchii). PeerJ.

[ref-3] Amadori M, Amalfitano J, Giusberti L, Fornaciari E, Luciani V, Carnevale G, Kriwet J (2019). First associated tooth set of a high cusped *Ptychodus* (Chondrichthyes, Elasmobranchii) from the Upper Cretaceous of northeastern Italy, and resurrection of *Ptychodus altior* Agassiz, 1895. Cretaceous Research.

[ref-4] Amadori M, Kindlimann R, Fornaciari E, Giusberti L, Kriwet J (2022). A new cuspidate ptychodontid shark (Chondrichthyes; Elasmobranchii), from the Upper Cretaceous of Morocco with comments on tooth functionalities and replacement patterns. Journal of African Earth Sciences.

[ref-5] Amadori M, Kovalchuk O, Barkaszi Z, Giusberti L, Kindlimann R, Kriwet J (2023). A diverse assemblage of *Ptychodus* species (Elasmobranchii: Ptychodontidae) from the Upper Cretaceous of Ukraine, with comments on possible diversification drivers during the Cenomanian. Cretaceous Research.

[ref-6] Antczak M, Ruciński MR, Stachacz M, Matysik M, Król JJ (2020). Diversity of vertebrate remains from the Lower Gogolin Beds (Anisian) of southern Poland. Annales Societatis Geologorum Poloniae.

[ref-7] Bengtson JA, Perrin WF, Wursig B, Thiewissen JGM (2002). Crabeater seal Lobodon carcinophaga. Encyclopedia of Marine Mammals.

[ref-8] Chatterjee S, Small BJ (1989). New plesiosaurs from the Upper Cretaceous of Antarctica, origins and evolution of the Antarctic biota. Geology Society Special Publication.

[ref-9] Dentzien-Dias PC, de Figueiredo AMQ, Horn B, Cisneros JC, Schultz CL (2012). Paleobiology of a unique vertebrate coprolites concentration from Rio do Rasto Formation (Middle/Upper Permian), Paraná Basin, Brazil. Journal of South American Earth Sciences.

[ref-37] Eriksson ME, Lindgren J, Chin K, Mansby U (2011). Coprolite morphotypes from the Upper Cretaceous of Sweden: novel views on an ancient ecosystem and implications for coprolite taphonomy. Lethaia.

[ref-10] Griesshaber E, Schmahl WW, Neuser R, Pettke T, Blüm M, Mutterlose J, Brand U (2007). Crystallographic texture and microstructure of terebratulide brachiopod shell calcite: an optimized materials design with hierarchical architecture. American Mineralogist.

[ref-11] Hamm SA (2017). First associated tooth set of *Ptychodus mammillaris* in North America, Pfeifer Shale Member (lower middle Turonian), Greenhorn Limestone. Transactions of the Kansas Academy of Science.

[ref-38] Hamm SA (2020). Stratigraphic, geographic and paleoecological distribution of the Late Cretaceous shark genus *ptychodus* within the Western Interior Seaway, North America. New Mexico Museum of Natural History & Science Bulletin.

[ref-12] Harries P, Ozanne CR (1998). General trends in predation and parasitism upon inoceramids. Acta Geologica Polonica.

[ref-13] Hattin DE (1975). Stratigraphy and depositional environment of Greenhorn Limestone (Upper Cretaceous) of Kansas. Kansas Geological Survey Bulletin.

[ref-39] Hornibrook NdeB, Brazier RC, Strong CP (1989). Manual of New Zealand permian to pleistocene foraminiferal biostratigraphy. New Zealand Geological Survey Paleontological Bulletin.

[ref-14] Hunt AP, Lucas SG (2021). The ichnology of vertebrate consumption: dentalites, gastroliths and bromalites. New Mexico Museum of Natural History and Science Bulletin.

[ref-15] Hunt AP, Lucas SG, Milàn J, Lichtig AJ, Jagt JWM, Sullivan RM, Lucas SG (2015). Vertebrate coprolites from Cretaceous chalk in Europe and North America and the shark surplus paradox. Fossil Record 4. New Mexico Museum of Natural History and Science Bulletin.

[ref-16] Jagt-Yazykova E, Mazurek D, Kędzierski M, Jagt JWM, Todes JP, Walaszczyk I, Todes JP (2022). Palaeoenvironments and biota of the Opole Cretaceous. Cretaceous of Poland and of adjacent areas. Field trip: Guides.

[ref-17] Jiménez-Berrocoso Á, Olivero EB, Elorza J (2006). New petrographic and geochemical insights on diagenesis and palaeoenvironmental stress in Late Cretaceous inoceramid shells from the James Ross Basin. Antarctica Antarctic Science.

[ref-18] Kauffman EG (1972). *Ptychodus* predation upon a Cretaceous *Inoceramus*. Palaeontology.

[ref-19] Kłapciński J, Teisseyre B (1981). Utwory kredy Górnej pomiędzy brzegiem a opolem. Geologia Sudetica.

[ref-20] Leblanc ARH, Mohr SR, Caldwell MW (2019). Insights into the anatomy and functional morphology of durophagous mosasaurines (Squamata: Mosasauridae) from a new species of *Globidens* from Morocco. Zoological Journal of the Linnean Society.

[ref-21] Mazurek D (2008). Paleoecology and biostratigraphy of Turonian strata (Late Cretaceous) of the Odra Quarry in Opole.

[ref-22] Niedźwiedzki R (2005). The paleobathymetry and paleogeographical distribution of the Upper Cretaceous selachians from the Middle Europe (SW Poland) and their relationship with North American assemblages. Journal of Vertebrate Paleontology.

[ref-23] Niedźwiedzki R, Kalina M (2003). Late Cretaceous sharks in the Opole Silesia region (SW Poland). Geologia Sudetica.

[ref-24] Pommery Y, Scheyer TM, Neenan JM, Reich T, Fernandez V, Voeten DFAE, Losko AS, Werneburg I (2021). Dentition and feeding in Placodontia: tooth replacement in *Henodus chelyops*. BMC Ecology and Evolution.

[ref-25] Purnell M, Darras LPG (2015). 3D tooth microwear texture analysis in fishes as a test of dietary hypotheses of durophagy. Surface Topography Metrology and Properties.

[ref-26] Qvarnström M, Niedźwiedzki G, Tafforeau P, Žigaite Ž, Ahlberg PE (2017). Synchrotron phase contrast microtomography of coprolites generates novel palaeobiological data. Scientific Reports.

[ref-27] Qvarnström M, Wernström JV, Piechowski R, Tałanda M, Ahlberg PE, Niedźwiedzki G (2019). Beetle-bearing coprolites possibly reveal the diet of a Late Triassic dinosauriform. Royal Society Open Science.

[ref-28] Qvarnström M, Fikáček M, Wernström JV, Huld S, Beutel RG, Arriaga-Varela E, Ahlberg PE, Niedźwiedzki G (2021). Exceptionally preserved beetles in a Triassic coprolite of putative dinosauriform origin. Current Biology.

[ref-29] Raguin E, Rechav K, Brumfeld V, Shahar R, Weiner S (2020). Unique three-dimensional structure of a fish pharyngeal jaw subjected to unusually high mechanical loads. Journal of Structural Biology.

[ref-30] Sasko DE, Dean MN, Motta PJ, Hueter RE (2006). Prey capture behavior and kinematics of the Atlantic cownose ray, *Rhinoptera bonasus*. Zoology.

[ref-31] Scheyer TM, Neenan JM, Renesto S, Saller F, Hagdorn H, Furrer H, Tintori A (2011). Revised paleoecology of placodonts—with a comment on “The shallow marine placodont *Cyamodus* of the central European Germanic Basin: its evolution, paleobiogeography and paleoecology” by C.G. Diedrich. Historical Biology.

[ref-32] Shimada K, Everhart MJ, Decker R, Decker PD (2010). A new skeletal remain of the durophagous shark, *Ptychodus mortoni*, from the Upper Cretaceous of North America: an indication of gigantic body size. Cretaceous Research.

[ref-33] Shimada K, Rigsby CK, Kim SH (2009). Partial skull of Late Cretaceous durophagous shark, *Ptychodus occidentalis* (Elasmobranchii: Ptychodontidae), from Nebraska, U.S.A. Journal of Vertebrate Paleontology.

[ref-34] Strong CP, Raine JI, Terezow M (2018). Key species of New Zealand fossil foraminifera: descriptions from “Manual of New Zealand Permian to Pleistocene Foraminiferal Biostratigraphy” by Hornibrook, Brazier and Strong, 1989.

[ref-35] Walaszczyk J (1992). Turonian through Santonian deposits of the Central Polish Uplands; their facies development, inoceramid paleontology and stratigraphy. Acta Geologica Polonica.

[ref-36] Yazykova E (2022). Kiedy miasto było morzem.

